# Deletion of Rap-phosphatases for quorum sensing control in *Bacillus* and its effect on surfactin production

**DOI:** 10.1186/s13568-023-01555-6

**Published:** 2023-05-27

**Authors:** Chantal Treinen, Lennart Biermann, Maliheh Vahidinasab, Kambiz Morabbi Heravi, Lars Lilge, Rudolf Hausmann, Marius Henkel

**Affiliations:** 1 Institute of Food Science and Biotechnology, Department of Bioprocess Engineering (150k), University of Hohenheim, Fruwirthstr. 12, 70599 Stuttgart, Germany; 2 Cellular Agriculture, TUM School of Life Sciences, Technical University of Munich, Gregor-Mendel-Str. 4, 85354 Freising, Germany; 3 Department of Molecular Genetics, University of Groningen, Nijenborgh 7, Groningen, 9747 AG The Netherlands

**Keywords:** Rap-phosphatases, *Bacillus subtilis*, Molecular process control, Quorum sensing, Surfactin lipopeptide

## Abstract

The complex regulatory network in *Bacillus*, known as quorum sensing, offers many opportunities to modify bacterial gene expression and hence to control bioprocesses. One target regulated by this mechanism is the activity of the P_*srfA*_ promoter, which is engaged in the formation of lipopeptide surfactin. It was hypothesised that deletion of *rapC*, *rapF* and *rapH*, encoding for prominent Rap-phosphatases known to affect P_*srfA*_ activity, would enhance surfactin production. Therefore, these genes were deleted in a *sfp*^+^ derivative of *B. subtilis* 168 with subsequent evaluation of quantitative data. Up to the maximum product formation of the reference strain *B. subtilis* KM1016 after 16 h of cultivation, the titers of the *rap* deletion mutants did not exceed the reference. However, an increase in both product yield per biomass *Y*_P/X_ and specific surfactin productivity *q*_surfactin_ was observed, without any considerable effect on the ComX activity. By extending the cultivation time, a 2.7-fold increase in surfactin titer was observed after 24 h for strain CT10 (Δ*rapC*) and a 2.5-fold increase for CT11 (Δ*rapF*) compared to the reference strain KM1016. In addition, *Y*_P/X_ was again increased for strains CT10 and CT11, with values of 1.33 g/g and 1.13 g/g, respectively. Interestingly, the effect on surfactin titer in strain CT12 (Δ*rapH*) was not as distinct, although it achieved the highest promoter activity (P_*srfA*_-*lacZ*). The data presented support the possibility of involving the quorum sensing system of *Bacillus* in bioprocess control as shown here on the example of lipopeptide production.

**Supplementary Information:**

The online version contains supplementary material available at 10.1186/s13568-023-01555-6.

## Introduction

**Fig. 1:**
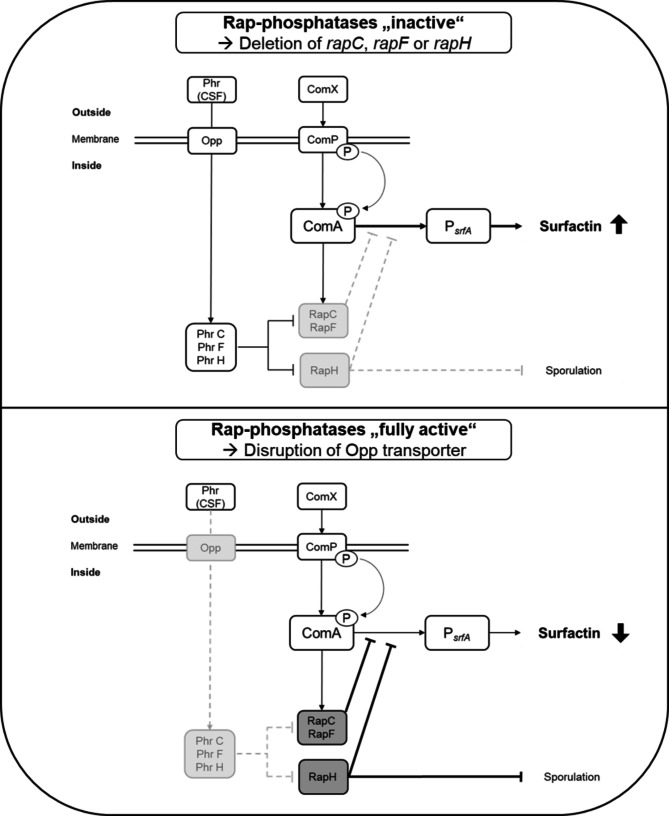
Schematic illustration of quorum sensing interactions in *Bacillus*. The figure is limited to the interactions that are addressed in this work. The upper part shows the influence of the deletion of Rap-phosphatases on the quorum sensing mechanism. The deletion is indicated in light gray. For simplification all Rap-phosphatases have been highlighted, although they were deleted individually in this work. In absence of the respective Rap-phosphatase, its inhibitory effect on the DNA-binding capacity of response regulator ComA is impaired (indicated by a dashed line). This means that the Rap-phosphatases should be “inactive” (strains CT10–CT12) and it is presumed that surfactin production is increased here. The lower part of the figure represents the negative control CT5, which carries a *oppA* deletion. Due to this deletion, the ABC transporter Opp should become non-functional. This is again highlighted in light gray. The Phr peptides responsible for Rap-phosphatase inhibition can therefore not be transported into the cell (indicated by a dashed line). Oppression of Rap-phosphatases should not be present in this case and therefore all Rap-phosphatases should be “fully active” (indicated in dark gray). For the negative control, it is assumed that surfactin production should be decreased. The hypotheses are supported by the literature, as described in the text. For an overview of molecular interactions, see Perego (2013)

The global quorum sensing system in *Bacillus* species presents major challenges and opportunities in bioprocess design due to its widespread involvement in various cellular mechanisms. These mechanisms include genetic competence and sporulation (Grossman 1995), biofilm formation (Špacapan et al. 2020) and also the activity of the *srfA* operon, responsible for the production of the lipopeptide surfactin (Nakano et al. 1991). To address the challenge of interactions between the quorum sensing regulatory network and surfactin production in bioprocessing, there are various approaches. For example, the native P_*srfA*_ promoter can be exchanged to uncouple surfactin production and the quorum sensing system (Coutte et al. 2010; Hoffmann et al. 2021; Willenbacher et al. 2016). Another option is to incorporate the quorum sensing system for lipopeptide production into the context of molecular process control, for example by modeling the kinetics of quorum sensing molecules (Treinen et al. 2021). However, in order to exploit the *Bacillus* quorum sensing system for bioprocess design, a deeper understanding of its components and their effects on the cells on both the qualitative and quantitative level is required. To this end, the interaction at the genetic level must first be assessed in more detail (Fig. 1). The pheromone ComX initiates a cascade, in which a phosphate group is transferred from ComP ~ P to ComA ~ P. This activates the response regulator ComA, which controls numerous processes in the cell, including a stimulatory effect on the surfactin promoter P_*srfA*_ (Comella and Grossman 2005; Magnuson et al. 1994; Nakano et al. 1991; Roggiani and Dubnau 1993). In addition to ComX, the competence and sporulation factor (CSF), also known as PhrC, is involved in the quorum sensing system. This 5-amino acid peptide influences both competence and sporulation in the cell depending on its concentration (Lazazzera et al. 1999; Solomon et al. 1996). Thereby, PhrC is oppressing RapC (Core and Perego 2003; Solomon et al. 1996), which belongs to the Rap protein family consisting of response-regulating aspartate phosphatases. To date, at least 11 Rap-phosphatases are known for *Bacillus subtilis* targeting various functions in the cell (Auchtung et al. 2006; Perego 2013). In one such interaction, RapC can impair the DNA-binding ability of ComA. This alters the expression of ComA-dependent genes independent of the phosphorylation state of ComA (Core and Perego 2003). RapF and RapH serve the same function, with RapH additionally being able to dephosphorylate Spo0F ~ P, a phosphotransferase involved in the sporulation initiation (Baker and Neiditch 2011; Smits et al. 2007). Due to this interaction with the response regulator ComA, the presence of certain Rap-phosphatases might minimize the promoter activity of P_*srfA*_ and thus negatively affect surfactin production. The molecular mechanism of Rap-phosphatases and their inhibitory Phr proteins has been extensively studied. However, most prior studies only qualitatively assessed the influence of Rap-phosphatases on ComA-dependent gene expression, leading to results that were not always comparable. For example, an increase in ComA-dependent *rapC* expression due to *rapC* deletion has been initially shown by Core and Perego (2003), but Bongiorni et al. (2005) found that a *rapC* deletion showed only a slight effect on the ComA-dependent *rapA* transcription. A deletion of both *rapC* and *rapF* showed a synergistic effect in the same study by Bongiorni et al. (2005). Contrarily, Auchtung et al. (2006) did not detect increased ComA-dependent expression after deletion of *rapF* and *rapK*, neither individually nor in combination with the deletion of *rapC*. In contrast to the aforementioned studies, this work aimed to investigate whether individual deletion of prominent Rap-phosphatases known to affect ComA, namely RapC, RapF, and RapH (Perego 2013) had an impact on surfactin titer and on specific surfactin productivity *q*_surfactin_. Additionally, ComX concentration was measured during the course of cultivation to study the quorum sensing mechanism in *Bacillus* from various angles and to derive further information on the deletions’ effects. Besides qualitative P_*srfA*_-*lacZ* measurements, the quantitative investigation offers deeper insight into the impacts of Rap-phosphatase-dependent deletions on the *Bacillus* quorum sensing system and its potential for strain improvement in industrial biotechnology. Therefore, this work can contribute to a deeper understanding of the quorum sensing system and furthers the potential to exploit it as a tool for future bioprocess design.

## Materials and methods

### Chemicals and standards

Unless otherwise stated, the chemicals used in this study were of analytical grade and purchased from Carl Roth GmbH & Co. KG (Karlsruhe, Germany). Reference standards from Sigma-Aldrich Laborchemikalien GmbH (Seelze, Germany) were used for HPTLC analysis, namely surfactin (≥ 98% purity) and glucose (≥ 99.5% purity).

### Microorganisms and strain maintenance

The strains used in this study were derived from *B. subtilis* JABs24 (Geissler et al. 2019) and are listed in Table 1. For ComX pheromone bioactivity assay, strain *B. subtilis* CT2 (Treinen et al. 2021) was used. Cells were conserved in lysogeny broth (LB), containing 15–25% (*v*/*v*) glycerol and stored at -80 °C.

**Table 1:** List of strains used for this study

Name	Genotype description	Reference
*B. subtilis*		
JABs24	*trp*+; Δ*manPA*; *sfp*+	(Geissler et al. 2019)^a^
KM1016	*trp*+; Δ*manPA*; *sfp*+; *amyE*::[P_*srfA*_-*lacZ, spc*]	(Hoffmann et al. 2021)
CT2	*trpC2*, Δ*comX*::*kan* *amyE*::[P_*srfA*_-*lacZ, spc*]	(Treinen et al. 2021)
BKE03770	*trpC2*; Δ*rapC*::*erm*	BGSC^b^ (Koo et al. 2017)
BKE37460	*trpC2*; Δ*rapF*::*erm*	BGSC^b^ (Koo et al. 2017)
BKE06830	*trpC2*; Δ*rapH*::*erm*	BGSC^b^ (Koo et al. 2017)
BKE11430	*trpC2*; Δ*oppA*::*erm*	BGSC^b^ (Koo et al. 2017)
CT5	*trp*+; Δ*manPA*; *sfp*+; P_*mtlA*_-*comK-comS*; Δ*oppA*;	This study
CT10	*trp*+; Δ*manPA*; *sfp*+; P_*mtlA*_-*comK-comS*; Δ*rapC*; *amyE*::[P_*srfA*_-*lacZ, spc*]	This study
CT11	*trp*+; Δ*manPA*; *sfp*+; P_*mtlA*_-*comK-comS*; Δ*rapF*; *amyE*::[P_*srfA*_-*lacZ, spc*]	This study
CT12	*trp*+; Δ*manPA*; *sfp*+; P_*mtlA*_-*comK-comS*; Δ*rapH*; *amyE*::[P_*srfA*_-*lacZ, spc*]	This study
*E. coli*		
JM109	[F´, *traD*36, *proA*^*+*^*B*^*+*^*lacI*^*q*^*Δ(lacZ)M*15], *Δ(lac-proAB), supE*44, *λ*^*−*^*gyrA96, recA*1, *relA*1, *endA*1, *thi, hsdR*17	Strain collection 150k^c^ (Yanisch-Perron et al. 1985)

^a^Kindly obtained from Dr. Josef Altenbuchner, Institute of Industrial Genetics, University of Stuttgart (Stuttgart, Germany)

^b^The *Bacillus* Genetic Stock Center (Columbus, USA)

^c^Department of Bioprocess Engineering (150k), Institute of Food Science and Biotechnology, University of Hohenheim (Stuttgart, Germany)

### Construction of mutant strains

Standard molecular techniques were applied for genetic engineering (Harwood and Cutting 1990). DNA and plasmid extractions were performed using the InnuPREP kits provided by Analytic Jena AG (Jena, Germany) as well as the QIAquick® Gel Extraction Kit (QIAGEN N.V., Venlo, Netherlands) following the instructor’s manual with minor adjustments by eluting extracted DNA with nuclease-free water. Primers and plasmids that were utilized for this work are presented in Table S1 and S2. Transformation was performed using inducible competence, as described by Rahmer et al. (2015). Therefore, an inducible competence cassette P_*mtlA*_-*comK-comS* was initially integrated into strain *B. subtilis* JABs24 using plasmids pHM30 and pJOE7361.1 (Motejadded and Altenbuchner 2007; Rahmer et al. 2015). In this way, competence was induced after addition of 0.5% mannitol (*w*/*v*). Selection pressure was achieved using the following chemicals: ampicillin (*amp*; 150 µg/µL), erythromycin (*erm*, 5 µg/mL), spectinomycin (*spc*, 100 µg/mL), histidine (*his*, 50 µg/mL). For gene deletion, the corresponding gene locus including approximately 1000 bp long flanking sites was amplified by PCR (Primer S1464–1465; S1467–1568; S1470–1471; S1560–1561) from the chromosomal DNA of *B. subtilis* knockout strains (BKE) provided by the *Bacillus* Genetic Stock Center (BGSC) (Koo et al. 2017). PCR was performed in a thermocycler (peqSTAR 96X VWR GmbH, Darmstadt, Germany) using the Q5® Hot Start High-Fidelity DNA Polymerase (New England Biolabs GmbH, Frankfurt am Main, Germany). Finally, the purified PCR fragment was used for transformation and selected using erythromycin. Successful deletion of *oppA* and *rap* genes was confirmed by PCR (Primer S1484; S1466; S1469; S1472; S1562) and Sanger sequencing was performed by Eurofins Genomics Germany GmbH (Ebersberg, Germany) using Primer S1567–S1574. For the subsequent removal of the erythromycin resistance gene, flanking *lox* sites were used in combination with Cre recombinase encoded on plasmid pJOE6732.1 (Morabbi Heravi and Altenbuchner 2018). All mutant strains were confirmed for successful *erm* removal by phenotype verification, PCR (Primer S1464–1465; S1467–1568; S1470–1471; S1560–1561) and Sanger sequencing (Primer S1567–S1574). For strains CT10–CT12, *lacZ* fusion with P_*srfA*_ was integrated into the *amyE* locus, using plasmid pKAM446 as previously described by Hoffmann et al. (2021). Successful integration was checked by phenotype verification and PCR (Primer S1637–S1638) and sanger sequencing confirmed the absence of point mutations (Primer S1639–S1640).

### Media

LB medium consisted of 10 g/L tryptone, 5 or 10 g/L NaCl and 5 g/L yeast extract, and in case of LB agar plates additionally 15 g/L bacteriological agar (Bertani 1951). For shake flask cultivation, an optimized mineral salt medium (MSM) was used (Willenbacher et al. 2015), with a glucose concentration of 8 or 40 g/L. Cultivations were carried out at pH 7.0 and media components were sterilized using an autoclave (15 min, 1 bar, 121 °C) or a sterile filter (0.22 μm). For cultivation of tryptophan auxotrophic strains, tryptophan (50 µg/mL) was supplemented.

### Cultivation conditions

All cultivation experiments were conducted in baffled shake flasks at 120 rpm and 37 °C using incubator shakers (Newbrunswick^TM^/Innova® 44, Eppendorf AG, Hamburg, Germany). Precultures were prepared as described in Treinen et al. (2021) with relative filling volumes of 0.2 mL/mL (20%). In detail, preculture I was carried out in LB medium and incubated for 15–16 h. Preculture I was then used to inoculate preculture II, which was carried out in MSM and incubated for 12 h. Exponentially growing cells were used for inoculation of the main culture, which was performed in 1000 mL shake flasks using a relative filling volume of 0.1 mL/mL (10%) and an initial OD_600_ of 0.1.

### Sampling and sample analysis

Sampling was performed every 2, 4 or 6 h, starting from *t*_0_ = 0 h. After determination of the optical density, OD_600_ (Biochrom WPA CO8000, Biochrom Ltd., Cambridge, UK), 100 µL of cultivation broth was used for Miller assay (Miller 1972). The promoter activity of the surfactin promoter (P_*srfA*_-*lacZ*) was measured using the protocol described by Hoffmann et al. (2020). Cell-free supernatant was harvested after centrifugation at 10 min at 4816 *g* and 4 °C (Heraeus X3R, Thermo Fisher Scientific GmbH, Braunschweig, Germany) and stored at -20 °C. The cell dry weight (CDW) could be calculated from the OD_600_ as described by Geissler et al. (2019), using a self-determined conversion factor of 3.3 ± 0.6. All samples were further analyzed for surfactin production and partly for ComX activity and glucose depletion. Both, the glucose and the surfactin concentration were analyzed using High-Performance Thin-Layer Chromatography, HPTLC (CAMAG Chemie-Erzeugnisse und Adsorptionstechnik AG, Muttenz, Switzerland). The applied methods were performed as previously described (Geissler et al. 2017, 2019). Quantifications that were not possible with an application volume of ≤ 5 µL of undiluted sample were assessed as not detectable. ComX bioassay was performed as described in Treinen et al. (2021) with minor adjustments. In brief, reporter strain *B. subtilis* CT2 was used to determine ComX-dependent expression of *lacZ*. Therefore, 750 µL of a pre-grown culture of strain CT2 were mixed with 750 µL of cell-free supernatant and incubated for 3 h at 120 rpm and 37 °C. Since the strains under investigation also carry the P_*srfA*_-*lacZ* cassette, the resistance of strain CT2 against kanamycin was exploited. To prevent growth of the strains studied and thus bias of the results, kanamycin (5 µg/mL) was added to the mixture. The resulting β-galactosidase activity was measured with the Miller assay, following the protocol of Hoffmann et al. (2020). Miller units were calculated using Eq. 1.

1 $${\rm{MU = 1000}} \cdot \frac{{{\rm{O}}{{\rm{D}}_{{\rm{420nm}}}}{\rm{ - }}\left( {{\rm{1}}{\rm{.75 }} \cdot {\rm{O}}{{\rm{D}}_{{\rm{550nm}}}}} \right)}}{{{\rm{t}} \cdot {\rm{v}} \cdot {\rm{O}}{{\rm{D}}_{{\rm{600nm}}}}}}$$

The limit of quantification (LOQ) for ComX bioassay was determined by Treinen et al. (2021), resulting in LOQ = 42.7 MU for shake flask cultivation.

### Data analysis and process parameters

All cultivation experiments were performed as biological duplicates. The data were evaluated with means of product yield per biomass *Y*_P/X_ [g/g], the specific growth rate *µ* [1/h] and the specific productivity *q* [g/(g·h)] using the equations provided by Geissler et al. (2019). The maximum growth rate *µ*_max_ and the maximum specific surfactin productivity *q*_max_ represent the highest achieved values throughout the cultivation process. The overall growth rate *µ*_overall_ as well as the overall specific surfactin productivity *q*_overall_ refer to the entire cultivation period and were calculated at either CDW_max_ or P_max_, if not indicated otherwise. The presented plots were created using scientific graphing analysis software Sigma Plot (Systat Software Inc., San Jose, USA).

## Results

**Performance of deletion strains in comparison to* B. subtilis* KM1016 until P**_**max**_** of reference**.

In a first experiment, the strains KM1016, CT5 and CT10–CT12 were cultivated as duplicates in shake flasks and discussed until the maximum surfactin concentration of reference strain KM1016 was reached. The time-course of the cell dry weight CDW as well as the glucose consumption of the individual strains can be found in Figure S1 in the supplementary information. Interestingly, after 16 h of cultivation the CDW of strain CT5 with the deleted *oppA* was considerably higher with CDW_max_ = 5.7 ± 0.2 g/L, also reaching the highest measured growth rate of *µ*_max_ = 0.57 1/h (Table 2). In comparison, the reference strain KM1016 achieved a CDW_max_ of 2.5 g/L and a growth rate of *µ*_max_ = 0.43 1/h. The deletions strains CT10–CT12 yielded overall the lowest biomass titers, namely between 1.2 and 1.5 g/L.

**Table 2:** Overview of cultivation and process parameters up until the maximum surfactin concentration P_max_ was reached for reference strain KM1016 at t = 16 h

Parameter	KM1016 168 *sfp*^+^		CT5 Δ*oppA*		CT10 Δ*rapC*		CT11 Δ*rapF*		CT12 Δ*rapH*	
**X**_**max**_ **[g/L]**	2.5 ± 0.4	16 h	5.7 ± 0.2	16 h	1.4 ± 0.0	16 h	1.2 ± 0.0	16 h	1.5 ± 0.0	16 h
**P**_**max**_ **[mg/L]**	920.3 ± 65.1	16 h	31.8 ± 9.1	12 h	707.5 ± 109.4	16 h	627.9 ± 48.6	16 h	735.4 ± 31.9	16 h
**P**_***srfA***_ **-** ***lacZ***_**max**_ **[MU]**	197.2 ± 24.1	14 h	n.d.	n.d.	227.1 ± 3.5	16 h	259.2 ± 21.0	16 h	532.2 ± 47.0	14 h
**ComX**_**max**_ **[MU]**	308.5 ± 40.5	16 h	211.5 ± 9.3	14 h	331.2 ± 73.2	16 h	231.5 ± 57.9	14 h	281.8 ± 16.2	16 h
***Y***_**P/X**_ **[g/g] at P**_**max**_	0.68		0.02		0.95		0.98		0.94	
***µ***_**max**_ **[1/h]**	0.43		0.57		0.53		0.48		0.45	
***µ***_**overall**_ **[1/h] at X**_**max**_	0.27		0.32		0.27		0.24		0.27	
***q***_**max**_ **[g/(g·h)]**	0.18		0.01		0.15		0.15		0.25	
***q***_**overall**_ **[g/(g·h)] at P**_**max**_	0.04		0.002		0.06		0.06		0.06	

### Activity of surfactin promoter P_***srfA***_ upon t = 16 h

**Fig. 2:**
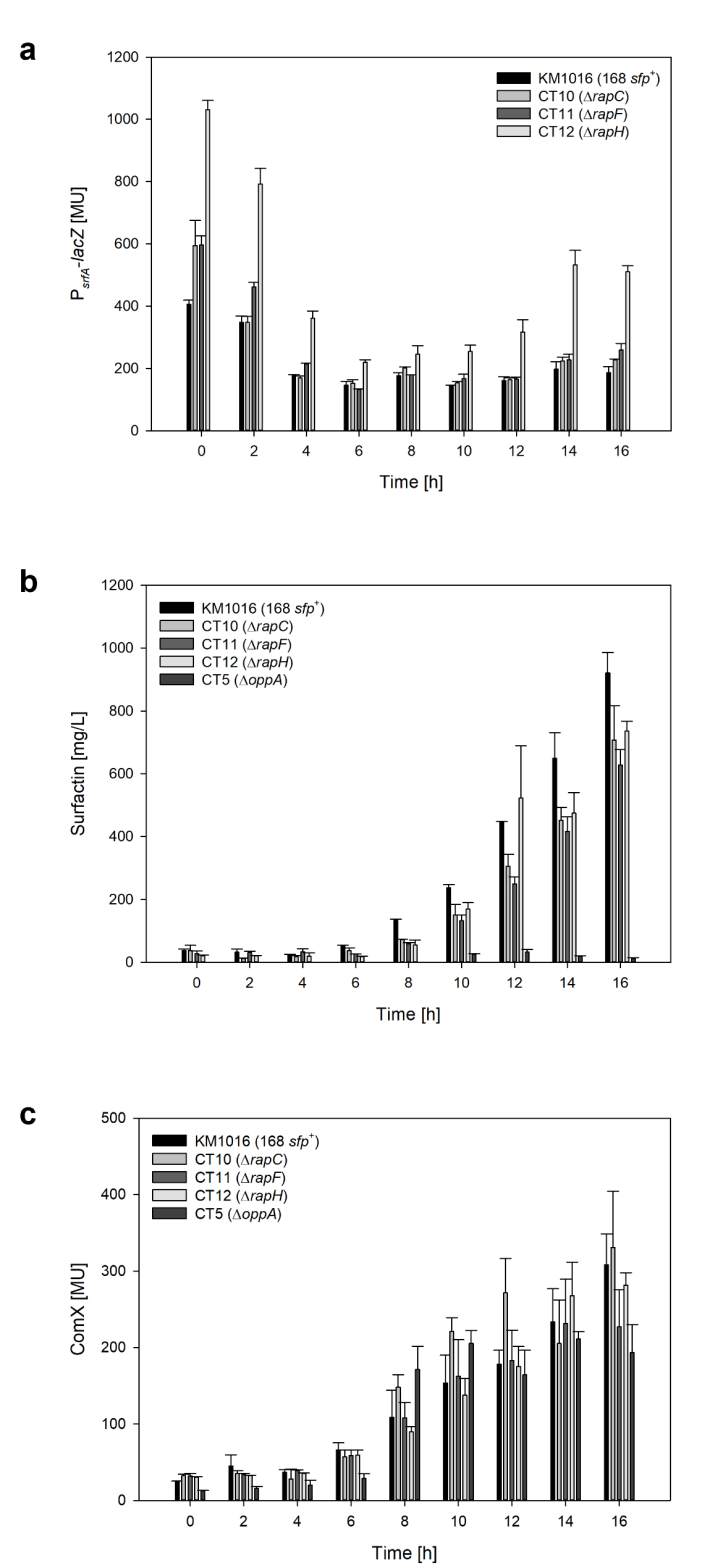
Bar charts of promoter activity P_*srfA*_-*lacZ* (**a**), surfactin concentration (**b**) and ComX activity (**c**) over the course of cultivation until the maximum surfactin concentration of reference strain KM1016 was reached at t = 16 h. LOQ = 42.7 MU for ComX bioassay, as determined in Treinen et al. (2021)

As seen in Fig. 2A, the activity of surfactin promoter P_*srfA*_ was especially high during the lag phase between t = 0–4 h. An explanation is the fact that after inoculation the formed β-galactosidase of the preculture was transferred to the main culture. At this point the corresponding OD_600_ was however still very low. Since the OD_600_ is included in the calculation of the Miller units in the denominator (Eq. 1), the value was initially high and decreased when cell growth started, and then increased again after fresh β-galactosidase has been produced. This has previously been observed in numerous publications, including Bongiorni et al. (2005), Hoffmann et al. (2021) and Treinen et al. (2021). For the evaluation of the data, the initial high points during lag phase were not taken into account. Hence, a maximum P_*srfA*_-*lacZ* of 197.2 ± 24.1 MU was reached after 14 h for reference strain KM1016. In comparison, P_*srfA*_-*lacZ* activities of strain CT10 and CT11 were slightly higher, reaching values of 227.1 ± 3.5 MU for CT10 and 259.2 ± 21.0 MU for CT11. Interestingly, the promoter activity observed for the deletion of *rapH* reached considerably higher values of 532.2 ± 47.0 MU after 14 h.

### Quantitative surfactin analysis and cultivation parameters upon t = 16 h

Looking at quantitative data, the highest surfactin titer was reached for the reference strain KM1016 with a P_max_ of 920.3 ± 65.1 mg/L after 16 h of cultivation (Fig. 2B). Although the promoter activities were in similar range or higher for the deletion mutants CT10–CT12, the surfactin titers were lower than the control strain. The maximum titers were 707.5 ± 109.4 mg/L surfactin for CT10, 627.9 ± 48.6 mg/L for CT11 and 735.4 ± 31.9 mg/L for CT12, all reached after 16 h of cultivation. Interestingly, the high P_*srfA*_ activity of RapH was not reflected in the surfactin concentration. However, looking at the maximum specific surfactin productivity *q*_max_ (Table 2), strain CT12 had the highest value of 0.25 g/(g·h). Also, with regards to the overall specific surfactin productivity *q*_overall,_ the mutant strains were exceeding the values of reference KM1016, reaching 0.06 g/(g·h) for CT10–CT12 and 0.04 g/(g·h) for KM1016. This trend was also evident in the product yield per biomass. The highest yield was achieved for strain CT11 with a *Y*_P/X_ of 0.98 g/g. Both CT10 and CT12 had comparable values of 0.95 g/g and 0.94 g/g respectively. As expected, the negative control CT5 for quantitative surfactin production showed the lowest surfactin concentration, with a P_max_ of 31.8 ± 2.1 mg/L after 12 h. This was associated with a low product yield per biomass of *Y*_P/X_ = 0.02 g/g and a specific surfactin productivity of *q*_overall_ = 0.002 g/(g·h) (Table 2).

### ComX activity upon t = 16 h

Besides the regulatory effect of Rap-phosphatases on the response regulator ComA and thus on the P_*srfA*_ activity, the signaling molecule ComX is also important for the production of surfactin. To investigate this part of the quorum sensing mechanism, ComX activity was measured during the course of cultivation (Fig. 2C). ComX activities of the deletion mutants CT10 and CT12 were comparable to the reference strain KM1016 (308.5 ± 40.5 MU), when considering standard deviations. Maximum values of 331.2 ± 73.2 MU were reached for CT10 and 281.8 ± 16.2 MU for CT12. Strain CT11 showed a lower maximum of 231.5 ± 57.9 MU (Table 2). However, when looking at the overall time-course and not only the maximum value, small fluctuations in ComX levels were observed in all strains, but the trend remained the same.

### Influence of prolonged cultivation time on strain performance

**Fig. 3:**
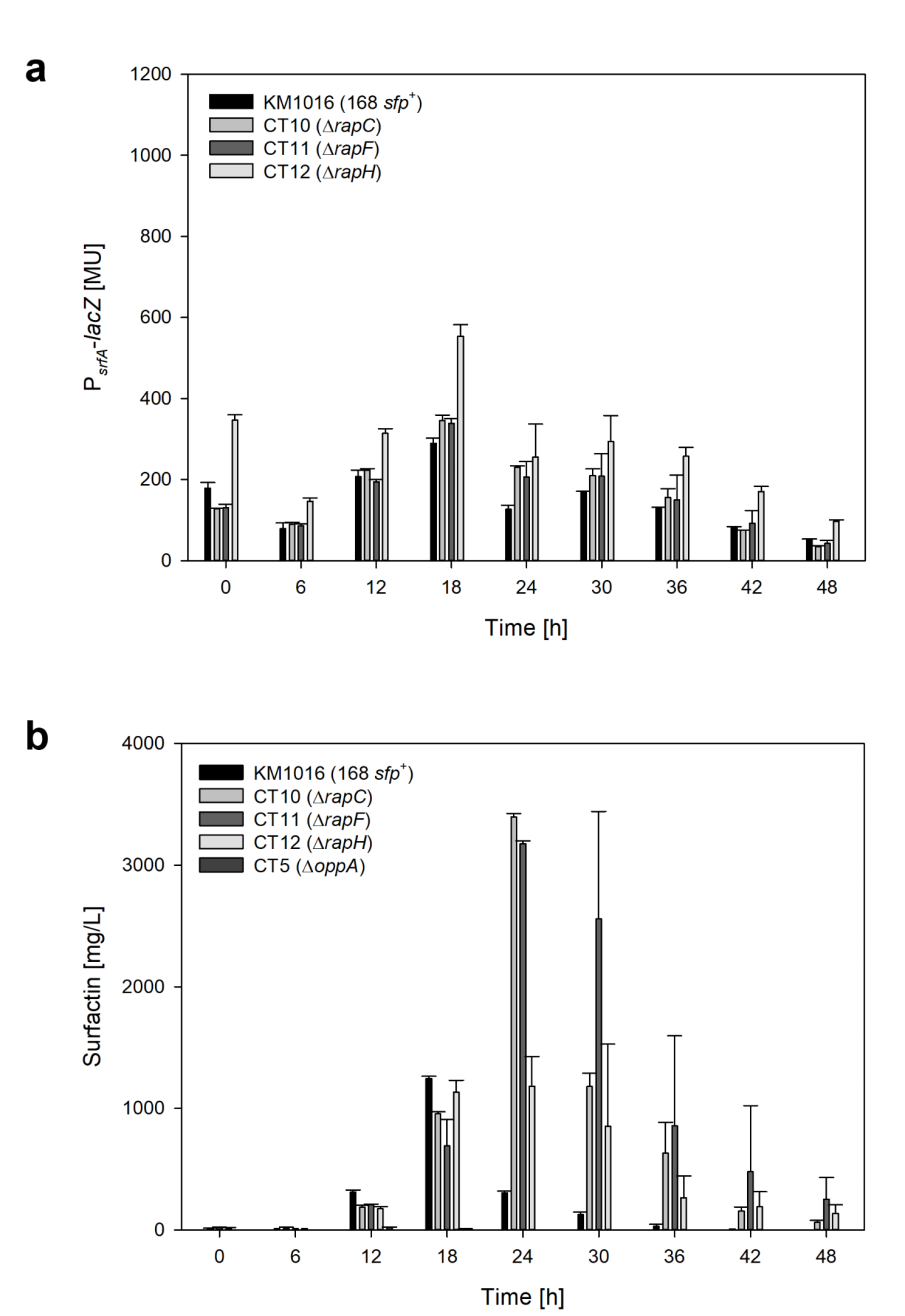
Bar charts of promoter activity P_*srfA*_-*lacZ* (**a**) and surfactin concentration (**b**) over the course of cultivation until t = 48 h

To answer the question whether a prolonged incubation period would increase the surfactin titer of the *rap* deletion strains, another cultivation was performed with the same experimental set-up but with longer sampling intervals to cover the full range of different growth stages up to 48 h of incubation. The time-course of the cultivation is shown in Figure S2 in the supplementary information and the analyzed data is provided in Table 3. In addition to the quantitative surfactin data, the promoter activity of P_*srfA*_-*lacZ* was analyzed, which is given in Fig. 3.

**Table 3:** Overview of cultivation and process parameters up until a cultivation time of t = 48 h. Calculations were performed at P_max_, if not indicated otherwise. Due to partially high standard deviations, additional technical replicates were performed for certain surfactin measurements and taken into account for the calculation of the mean values

Parameter	KM1016 168 *sfp*^+^		CT5 Δ*oppA*		CT10 Δ*rapC*		CT11 Δ*rapF*		CT12 Δ*rapH*	
**X**_**max**_ **[g/L]**	8.3 ± 0.0	24 h	5.5 ± 0.0	24 h	10.9 ± 0.5	48 h	8.7 ± 0.0	48 h	8.6 ± 2.1	24 h
**P**_**max**_ **[mg/L]**	1245.4 ± 18.1	18 h	23.8 ± 0.7	12 h	3395.2 ± 29.8	24 h	3175.0 ± 24.0	24 h	1181.8 ± 245.0	24 h
**P**_***srfA***_ **-** ***lacZ***_**max**_ **[MU]**	289.7 ± 13.0	18 h	n.d.	n.d.	346.2 ± 13.4	18 h	338.9 ± 11.6	18 h	553.8 ± 28.5	18 h
***Y***_**P/X**_ **[g/g] at P**_**max**_	0.83		0.01		1.33		1.13		0.96*	
***q***_**max**_ **[g/(g·h)]**	0.08		0.002		0.12		0.11		0.11	
***q***_**overall**_ **[g/(g·h)]**	0.05		0.001		0.06		0.05		0.05*	

* Calculation of process parameters for strain CT12 was performed at t = 18 h. Here, comparable surfactin levels (1133.4 ± 97.3 mg/L) were achieved, taking into account the standard deviation, which also occurred for the OD_600_ measurement at t = 24 h (Figure S2)

### Activity of surfactin promoter P_***srfA***_ upon t = 48 h

The overall highest promoter activity of P_*srfA*_-*lacZ =* 553.8 ± 28.5 MU (Table 3) was again achieved by strain CT12. However, with increasing cultivation time, a decrease in promoter activity was observed in all strains after reaching a maximum at 18 h of cultivation (Fig. 3A). Although strains CT10 and CT11 showed a comparable maximum to reference strain KM1016, an almost 2-fold higher P_*srfA*_ activity was still detected after the decrease at t = 24 h with 230.7 ± 3.1 MU (CT10) and 206.4 ± 37.9 MU (CT11), compared with 126.8 ± 10.1 MU for strain KM1016. At the end of the cultivation, the promoter activities were decreased to 50.7 ± 2.5 MU for the reference strain, 35.0 ± 2.5 MU for CT10 and 42.4 ± 7.5 MU for CT11. Again, CT12 stood out as the promoter activity was higher compared to all other strains with a remaining 97.0 ± 3.7 MU at t = 48 h.

### Quantitative surfactin analysis and cultivation parameters upon t = 48 h

Upon 18 h of cultivation, the surfactin production corresponded to the aforementioned results, although having overall higher titers (Fig. 3B). The highest surfactin concentration, P_max_ = 1245.4 ± 18.1 mg/L, was again reached for the reference strain KM1016, followed by CT12 (1133.4 ± 97.3 mg/L) and CT10 (956.0 ± 15.3 mg/L), while CT11 (691.9 ± 215.4 mg/L) had the lowest value. However, when the incubation time was extended, the surfactin levels of both CT10 and CT11 surpassed the P_max_ of KM1016. Strain CT10 achieved a P_max_ = 3395.2 ± 29.8 mg/L and CT11 a P_max_ = 3175.0 ± 24.0 mg/L, thereby increasing the levels of surfactin by 2.7 and 2.5-fold respectively. It is further noticeable that the product yield per biomass for both CT10 (*Y*_P/X_ = 1.33 g/g) and CT11 (*Y*_P/X_ = 1.13 g/g) increased considerably (Table 3). After reaching its maximum, surfactin concentration of reference strain KM1016 decreased starting from t = 24 h as typically described for *B. subtilis* cultures with high glucose concentration (as discussed in Treinen et al. (2021)). The previously postulated hypothesis that the balance is at a disadvantage of surfactin production (Klausmann et al. 2021; Treinen et al. 2021) is supported by the concomitant decline in P_*srfA*_ activity. This decrease in surfactin concentration is also shown for the mutant strains, but at a later time point, specifically at t = 30–36 h. Curiously, although strain CT12 again showed the overall highest promoter activity, this strain did not display a distinct increase in surfactin titer. Rather, a concentration was achieved that was at the same level as the reference strain with a P_max_ = 1181.8 ± 245.0 mg/L at t = 24 h (Table 3). Also, the product yield per biomass for strain CT12 (*Y*_P/X_ = 0.96 g/g) was only moderately higher compared to the reference (*Y*_P/X_ = 0.83 g/g) in the second experiment and not as prominent as for strains CT10 and CT11. With respect to specific surfactin productivity, only strain CT10 showed a marginally higher *q*_overall_ = 0.06 g/(g·h) compared to the reference KM1016 with a *q*_overall_ of 0.05 g/(g·h). Although all three deletion mutants (CT10 – CT12) displayed higher *q*_max_ values between 0.11 and 0.12 g/(g·h), compared to 0.08 g/(g·h) for strain KM1016, differences were not as evident as previously observed for strain CT12 (Table 2).

## Discussion

The Rap-phosphatases RapC, RapF and RapH, which are known to affect response regulator ComA, were deleted in the laboratory production strain JABs24 derived from *B. subtilis* 168. This allowed to assess the deletion of quorum sensing elements as a technique for molecular process control. The key outcomes of this study are listed in the following:

- Quantitative surfactin analysis to determine the impact of individual *rapC*, *rapF* and *rapH* deletions.
- Promoter activity P_*srfA*_ increased particularly for *rapH* deletion, whereas surfactin titers were not as strongly affected.
- Increased product yield per biomass *Y*_P/X_ and specific surfactin productivity *q* for deletion mutants.
- Increase in surfactin titer for *rapC* and *rapF* deletion only visible after prolonged incubation period.
- Deletion of Rap-phosphatases did not have any appreciable effect on ComX activity.

Additionally, a negative control was constructed by deleting *oppA*, which is important for a correct function of oligopeptide ABC transporter Opp. Considering that this strain was used as a reference for a low surfactin production, an additional P_*srfA*_-*lacZ* was not further investigated. But it is not only the Rap-phosphatases that influence the activity of ComA. Conversely, the expression of *rapC* as well as *rapF* is also regulated via ComA ~ P and thus induced by ComX (Comella and Grossman 2005). This led to the hypothesis in a previous study by Treinen et al. (2021) that high ComX levels during cultivation of *Bacillus* strains might negatively influence the surfactin production in form of a negative feedback loop. In turn, a deletion of the mentioned Rap-phosphatases could provide a checkpoint for strain improvement and the absence of the counteracting proteins could increase both surfactin titer and specific surfactin productivity *q*_surfactin_ (Fig. 1). At the same time, it is hypothesised that ComX concentration should remain constant or unaffected.

### Influence of ***rap*** deletion on P_***srfA***_ activity

Not considering the initial high values in the lag phase due to the transfer of the preculture, the maximal measured promoter activity of reference strain KM1016 was comparable to literature data. Here, a P_*srfA*_-*lacZ* of 197.2 ± 24.1 MU was obtained which is in agreement with Hoffmann et al. (2021), where about 200 MU were achieved with the same strain and a filling volume of 10% in shake flasks. In contrast, the deletion strains CT10 and CT11 deviated only moderately from the reference values. This was not unexpected as previous findings showed that a deletion of these Rap-phosphatases did not always lead to a large increase in promoter activity (Auchtung et al. 2006; Bongiorni et al. 2005; Core and Perego 2003). However, the deletion strain CT12 showed considerably higher promoter activities than the reference strain KM1016 in both conducted cultivations. In detail, a 2.7-fold increase was achieved at t = 14 h for cultivation 1 (Fig. 2A) and a 1.9-fold increase at t = 18 h for cultivation 2 (Fig. 3A). This was in contrast to a study of Hayashi et al. (2006) who stated that although overexpression of RapH resulted in a lower expression of *srfA*, only a marginal effect could be observed after the deletion of the aforementioned Rap-phosphatase in comparison to the control strain *B. subtilis* OSM100. The question now arises as to why the deletion of RapH stood out in comparison to RapC and RapF. In this context, it should be mentioned that RapH takes on a special role, since not only the response regulator ComA, but also sporulation is affected by dephosphorylation of Spo0F ~ P, which is required for sporulation initiation (Smits et al. 2007). Due to its influence on the DNA binding activity of ComA (Smits et al. 2007), a deletion of *rapH* would most likely also have an indirect effect on the expression of *rapC* and *rapF*, which in turn again influence ComA activity (Comella and Grossman 2005).Lazazzera et al. (1999) claimed that RapC negatively controls its own production and Core and Perego (2003) previously found that deletion of *rapC* positively affected transcription of *rapC*-*lacZ.* This suggests that a deletion of *rapH* might also positively influence transcription of *rapC* and *rapF*. A therefore increased RapC and RapF production may have masked the effect of the *rapH* deletion, keeping the surfactin titer in a similar range as the reference. The assumption that active Rap-phosphatases could mask the effect of *rapH* deletion was previously postulated in a study by Smits et al. (2007).

### Deletion strains showed higher ***Y***_P/X_ and ***q***_overall_ after reaching ***P***_***max***_ of reference

Reference strain KM1016 achieved the overall highest surfactin titer after 16 h during the first cultivation with a concentration of P_max_ of 920.3 ± 65.1 mg/L (Table 2). This was in range of surfactin titers typically measured for JABs24 in shake flask cultivations, as shown by Geissler et al. (2019), with 1147.03 mg/L surfactin at CDW_max_ after 21 h. However, the obtained process parameters such as *Y*_P/X_ and specific surfactin productivity *q*_overall_ were higher for the deletion strains CT10–CT12, while biomass titers were overall lower compared to the reference KM1016. Accordingly, the productivity per cell of the mutant strains was higher and thus supported the hypothesis that a deletion of Rap-phosphatases might positively influence surfactin production. The differences in achieved CDW_max_ were even more drastic for negative control Δ*oppA.* This indicated a trend that as surfactin production increased, the biomass production was lowered. This was also observed in a study by Vahidinasab et al. (2020) in which strain BMV12, with a deleted *srfA* operon showed higher OD_600_ values compared to the strains in which the operon was active. This suggests the possible explanation, that if surfactin production is low or absent, more metabolic energy would be available for biomass growth. Conversely, this could explain why strains CT10–CT12 achieved a lower CDW_max_ after 16 h than the reference strain KM1016 considering the higher *q*_surfactin_ for the mutant strains.

### Deletion of Rap-phosphatases only slightly affected ComX level

In Treinen et al. (2021), it was found that specific surfactin productivity *q*_surfactin_ did not increase linearly with the ComX activity. Since ComA ~ P also affects the expression of *rapC* and *rapF* (Comella and Grossman 2005), it was suggested that high ComX activity leads to high RapC and RapF concentration, which in turn lowers surfactin titer and surfactin productivity (Treinen et al. 2021). Conversely, this would imply that deletion of these Rap-phosphatases would abolish or attenuate this negative feedback and that, while ComX levels would remain constant, productivity as well as surfactin titer would increase. The ComX activities of the deletion strains were in a comparable range with the reference KM1016 or even lower. Furthermore, the obtained values were consistent with a previous study (Treinen et al. 2021), in which maximum ComX activities of 357.3 ± 41.9 MU were measured for strain *B. subtilis* DSM10^T^ in shake flask cultivations with 40 g/L glucose. Nonetheless, all deletion mutants showed higher overall specific surfactin productivities *q*_overall_. This supported the hypothesis already formulated in Treinen et al. (2021) and addressed again here. It also showed that the differences in measured promoter activity P_*srfA*_-*lacZ* may have been in fact due to the deletion of the Rap-phosphatases and not due to the differences in ComX levels.

### Negative control Δ***oppA*** showed low surfactin concentrations

A crucial difference to the mutants with deletion of *rap* is that in the Δ*oppA* variant no Phr, the antagonist of Rap, should be taken up into the cell (Lazazzera et al. 1997). Therefore, all Rap-phosphatases should be fully active in this strain (Fig. 1), as previously suspected by Auchtung et al. (2006), who found low P_*srfA*_-*lacZ* activity in a Δ*opp* mutant. Consistent with this, low surfactin titers and consequently low specific surfactin productivities were obtained with the negative control CT5. Since the ABC transporter Opp is affected by the deletion of *oppA*, this strain has a special role as further effects on the cell might occur that are not considered in the context of this study (Lazazzera et al. 1997).

### Prolonged cultivation time increased surfactin titer in Δ***rapC*** and Δ***rapF*** mutant

After prolonging the cultivation time, it was observed, that strains CT10 and CT11 exhibited elevated surfactin titers and product yield per biomass *Y*_P/X_. However, the specific surfactin productivities *q*_surfactin_ were at the same level or even lower. This can be explained by the fact that although the mutants showed increased product titers, these were only reached at a later stage of cultivation. Future process design should therefore consider whether the focus is on productivity or yield. One explanation for the fact that the effects on surfactin titer are now noticeable could be that the stationary phase has not yet been reached in the previous experiment, although it is known that surfactin production takes place in the late exponential to early stationary phase (Ongena and Jacques 2008). Especially in cultivations with low glucose concentrations this growth phase hardly occurs because the cell growth stops immediately upon glucose depletion, as seen in an aerobic cultivation of *B. subtilis* JABs24 with 1% glucose (*w*/*v*) (Geissler et al. 2019). In a similar manner, the deletion mutants were cultured in mineral salt medium containing 8 g/L glucose during preliminary experiments (Figure S3), and a decrease in CDW was observed after 16–20 h of cultivation without entering the stationary phase. At the same time, surfactin remained in a comparable range of approximately 900–1000 mg/L without a noticeable effect of the *rap* deletions. In the previously discussed studies, the medium of choice was either Schaeffer’s sporulation medium (Bongiorni et al. 2005; Core and Perego 2003; Smits et al. 2007) or a mineral salt medium with 10 g/L glucose (Auchtung et al. 2006). The amount of carbohydrate source and the associated growth behaviour of *B. subtilis* could therefore explain the not always knowledgeable effect of *rap* deletions observed so far. This assumption is strengthened by a study of Sun et al. (2018) who investigated the influence of *rapC* deletion on bacillomycin D production in *B. amyloliquefaciens* using 20 g/L glucose. There, a 1.5-fold increase in bacillomycin D from 240.7 ± 18.9 mg/L to 360.8 ± 30.7 mg/L was observed with prolonged cultivation, after the titer initially remained comparable. A possible influence of the chosen medium on the results was also mentioned in the study by Auchtung et al. (2006), who pointed also to the various promoters tested as a further explanation for inconsistent effects.

To exploit quorum sensing for process control, it is essential to understand the individual processes within the cell. In this study, it was hypothesised that deletion of Rap-phosphatases RapC, RapF and RapH would increase surfactin titer and specific surfactin productivity *q*_surfactin_. To the best of our knowledge, this is the first time that quantitative data on surfactin concentration have been collected in this context. Up to the P_max_ of the reference KM1016, all three deletion mutants showed an increased product yield per biomass *Y*_P/X_ and *q*_overall_ with consistent ComX levels. With prolonged cultivation time, the surfactin titer could even be enhanced by 2.7-fold for CT10 (Δ*rapC*) and 2.5-fold for CT11 (Δ*rapF*), which further supports the previously established hypothesis. The insights into surfactin production at the quantitative level obtained in this study contribute to the integration of the quorum sensing mechanism into process control instead of perceiving it as a constraint. For instance, incorporating a model and thus combining the influence of ComX and Rap-phosphatases on surfactin production could be used in the scope of molecular process control. At the genetic level, this study lays the groundwork to investigate other Rap-phosphatases following the same protocol or to create combinatorial mutants to examine a possible masking effect. Since metabolic regulation in *Bacillus* is very extensive, it would also be interesting to study the influence of *rap* deletion in relation to other parts of the system. For example, with respect to other lipopeptides produced by *Bacillus*, this study can be used as an example to gain deeper insights into the broader spectrum of the regulatory mechanism and further exploit its potential.

## Electronic supplementary material

Below is the link to the electronic supplementary material.

Supplementary Material 1

## Data Availability

All discussed data have been included into the manuscript or the supplementary material. Please turn to the corresponding author for all other requests.

## List of Abbreviations

CDW: Cell dry weight
CSF: Competence and sporulation factor
MSM: Mineral salt medium
MU: Miller units
Opp: Oligopeptide ABC transporter
Rap: Response regulator aspartate phosphatase
CT5: Δ*oppA*
CT10: Δ*rapC*
CT11: Δ*rapF*
CT12: Δ*rapH*
