# Supplementary material for: Deletion of Rap-phosphatases for quorum sensing control in Bacillus and its effect on surfactin production
Source: AMB Express. 2023 May 27;13:51. doi: 10.1186/s13568-023-01555-6 (PMC10224909; doi:10.1186/s13568-023-01555-6)
Supplement: Supplementary file 1 — Supplementary Material 1 [file 13568_2023_1555_MOESM1_ESM.pdf]

Supplementary Information

Deletion of Rap-phosphatases for quorum sensing control in *Bacillus* and its effect on surfactin production

Chantal Treinen[a,b], Lennart Biermann[a], Maliheh Vahidinasab[a], Kambiz Morabbi Heravi[a], Lars Lilge[c], Rudolf Hausmann[a] and Marius Henkel[b]*

[a]Institute of Food Science and Biotechnology, Department of Bioprocess Engineering (150k), University of Hohenheim, Fruwirthstr. 12, 70599 Stuttgart, Germany

[b]Cellular Agriculture, TUM School of Life Sciences, Technical University of Munich, Gregor-Mendel-Str. 4, 85354 Freising, Germany

[c]Department of Molecular Genetics, University of Groningen, Nijenborgh 7, 9747 AG Groningen, The Netherlands

***Corresponding author:**	Marius Henkel
Address:	Gregor-Mendel-Str. 4, 85354 Freising, Germany
Phone:	+49 8161 71 5130
Email:	marius.henkel@tum.de

Authors' email addresses:

chantal.treinen@tum.de, lennart.biermann@uni-hohenheim.de, vahidin@uni-hohenheim.de, kambiz.morabbi@gmail.com, l.lilge@rug.nl, rudolf.hausmann@uni-hohenheim.de, marius.henkel@tum.de

Overview of plasmids and primers used for this study

Table S1 List of plasmids used for this study.

Name	Properties or inserts	Reference
pHM30	*ori*$_{pUC18}$, *bla*, *‘hisF-hisI’*, s*pc*^{R}, *‘yvcA-yvcB’*	(Motejadded and Altenbuchner 2007)
pJOE7361.1	*ori*$_{pUC18}$, *bla*, *‘yvcB*-P$_{mtlA}$*-comK-comS-hisI-hisF’*	(Rahmer et al. 2015)
pJOE6732.1	*ori*$_{pUC18,}$ *spc*^{R}, *amp*^{R}, P$_{xylAB}$-P1-*cre*, instable vector (*B. subtilis*)	J. Altenbuchner (unpublished) (Morabbi Heravi and Altenbuchner 2018)
pKAM446	*ori*$_{pUC18}$*, bla*, *rop*, *ermC*, *amyE′*-[*ter*-P$_{srfAA}$-*lacZ*, *spc*^{R}]- *′amyE*	(Hoffmann et al. 2021)

Table S2 List of oligonucleotides used for this study. S1637 – S1640 are according to Hoffmann et al. (2021). Primers were obtained from Eurofins Genomics Germany GmbH (Ebersberg, Germany).

Name	Sequence (5' → 3')	Application
S1384	CCAATGAAGGTGCGGAAGTC	Confirmation of P_{mtlA}-*comK*-*comS* cassette integration into chromosome
S1385	CCTCACATCTTCGTCAGGAAC	
S1386	CGCCATTCTGAGATTGAAG	Sequencing of P_{mtlA}-*comK*-*comS* cassette integration into chromosome
S1387	ACACCATGTACTCTCTCG	
S1464	GAGTCATCCGCTCAAGCT	*oppA* amplification
S1465	ACCTTGAGAACATCGGCG	
S1466	ATCATCTGTTCCGAACCAGTG	confirmation of Δ*oppA*
S1467	GACAGATAAAGTGGCTTCCG	*rapC* amplification
S1468	GTCAATCCGTCCTTCGTTG	
S1469	ACGGAGCGGTTCTGATATTACCG	confirmation of Δ*rapC*
S1470	CATAGTCTGTTGCTGATTGCG	*rapF* amplification
S1471	AGAACCGCTTTCTCACTCC	
S1472	AGCACCTCGTGTCATGGC	confirmation of Δ*rapF*
S1560	TAGAATGCCTTGATGTTGCTGG	*rapH* amplification
S1561	CCTTCGTAGCCATACTCTCC	
S1562	GATACGATTTCACAGCGGAC	confirmation of Δ*rapH*
S1484	ATCGTGGAATACGGGTTTGC	confirmation of *erm* integration
S1567	GTTCACTCTATTGTTACAGC	*oppA* sequencing primer
S1568	TCAATTTCAGGCGGAAGC	
S1569	ACAGCAATAGCATACAGGG	*rapC* sequencing primer
S1570	GTCAGGTTACGGAACTTCT	
S1571	GACATACACTGCTCACGC	*rapF* sequencing primer
S1572	GCATTTCCACTCTCTTCAAG	
S1573	GGAGATGCTTTATCAGTTTG	*rapH* sequencing primer
S1574	CGGAGTTTGTCAGGGTTG	
S1637	GCGTAATAGACTTTCAGGCGT	Confirmation of P_{srfA}-*lacZ* integration into *amyE*
S1638	GCTTCATCCACCACATACAGG	
S1639	AGCCGCTGAAGAATATGG	Sequencing of P_{srfA}-*lacZ* integration into *amyE*
S1640	CGTAATGGGATAGGTCAC	

Time-course of CDW and glucose during shake flask cultivation (t = 16 h)

KM1016
(168 *sfp*+)

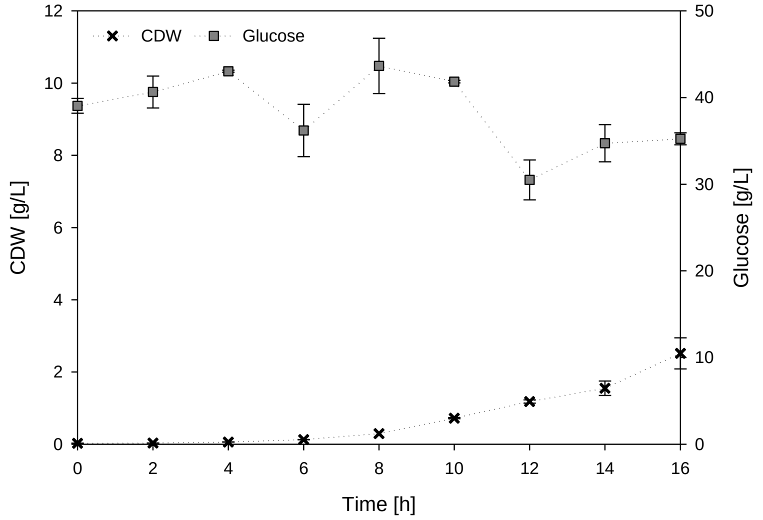

CT5
(Δ*oppA*)

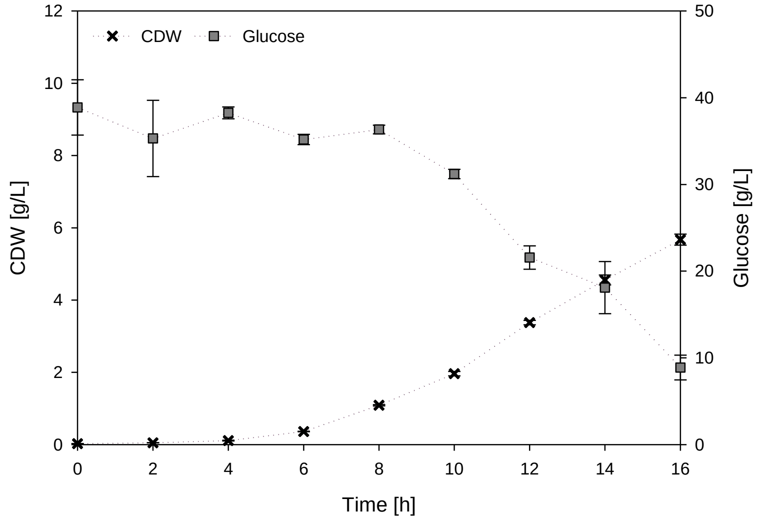

CT10
(Δ*rapC*)

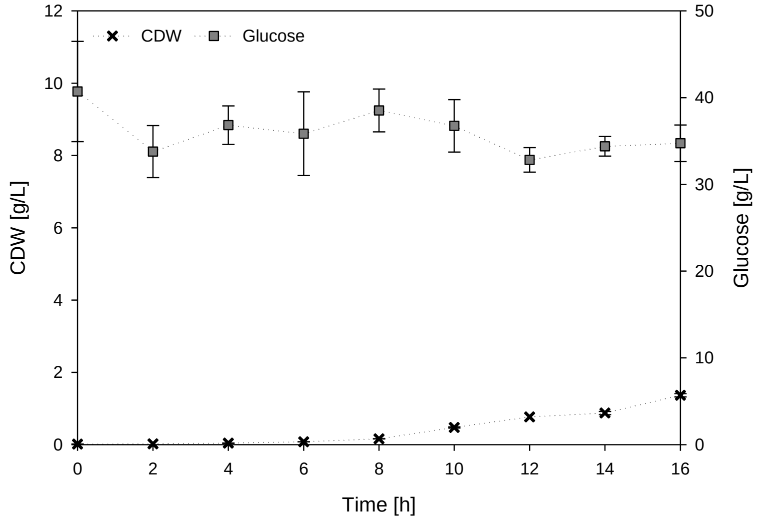

CT11
(Δ*rapF*)

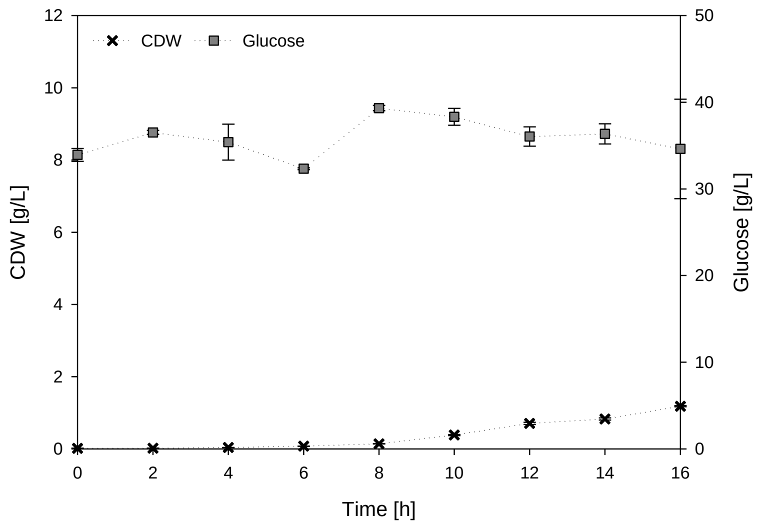

CT12
(Δ*rapH*)

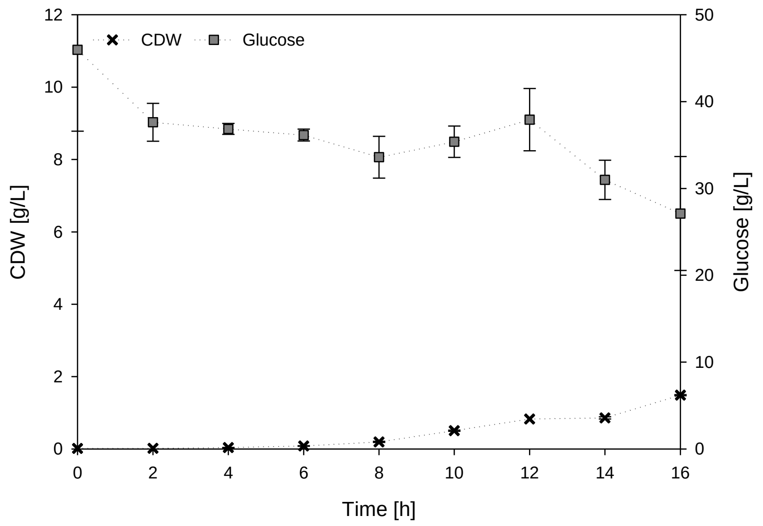

Fig. S1 Time-course of shake flask cultivation of *B. subtilis* mutation strains employing 40 g/L glucose until t = 16 h. Plotted are CDW formation (black cross) and glucose consumption (gray square) against the cultivation time.

Time-course of CDW during shake flask cultivation (t = 48 h)

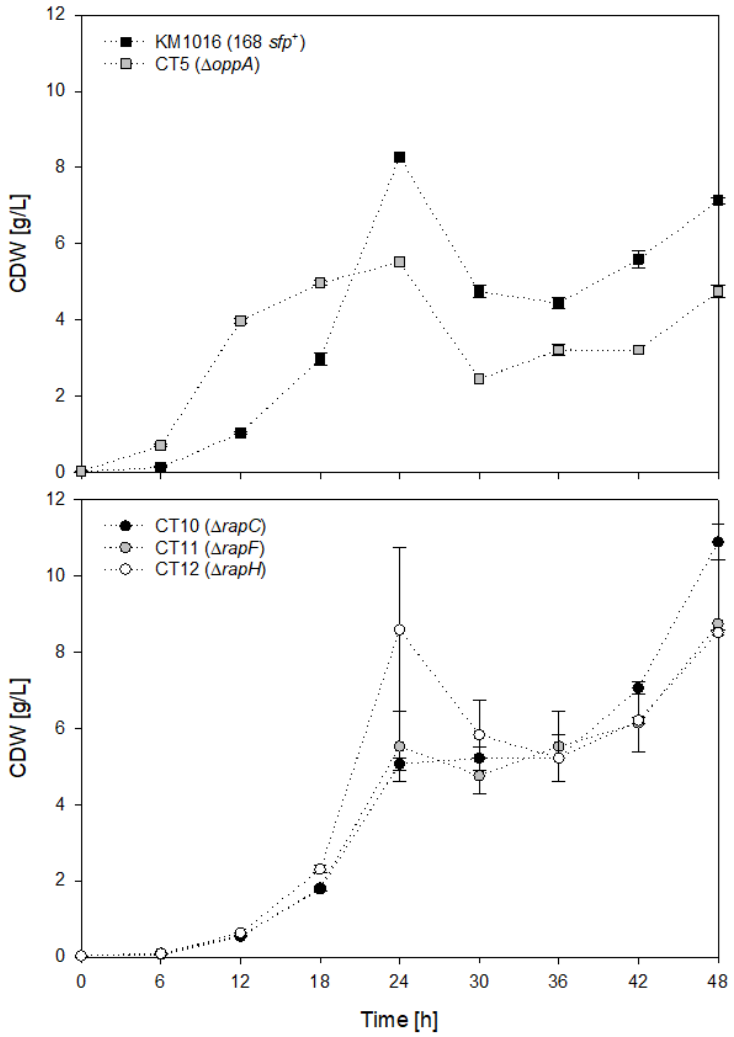

Fig. S2 Time-course of shake flask cultivation of *B. subtilis* mutation strains employing 40 g/L glucose until t = 48 h. Plotted are CDW formation for the strains KM1016 (black square) and CT5 (gray square) in the upper diagram and CT10 (black circle), CT11 (gray circle) and CT12 (white circle) in the lower diagram.

Time-course of CDW and surfactin during shake flask cultivation (8 g/L glucose)

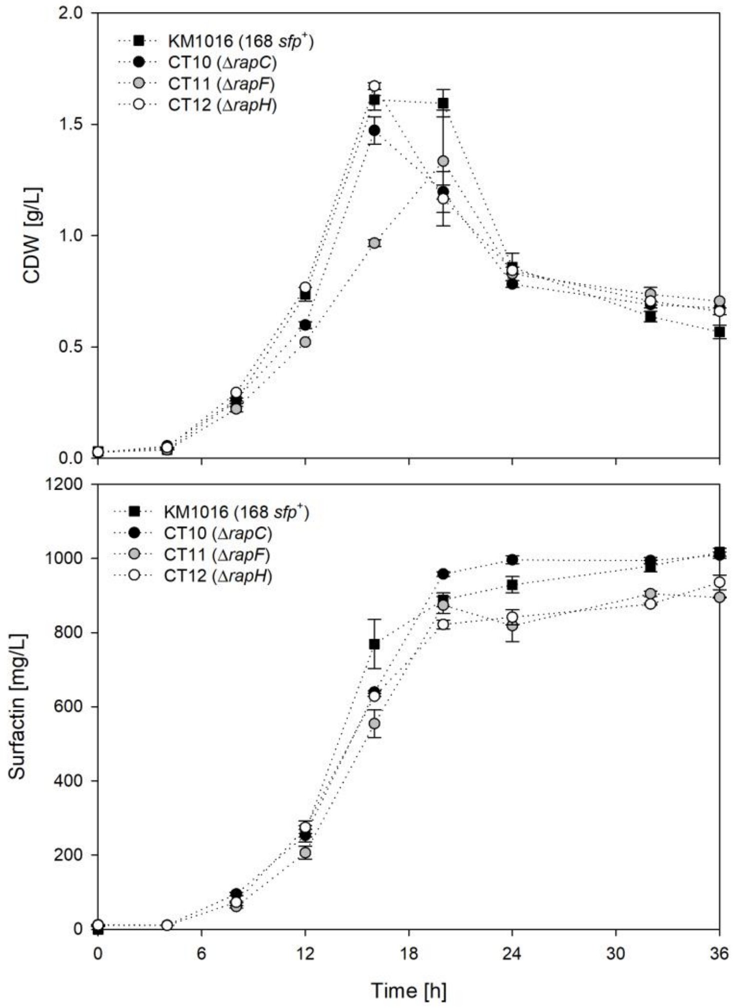

Fig. S3 Time-course of shake flask cultivation of *B. subtilis* mutation strains employing 8 g/L glucose until t = 36 h. Plotted are CDW formation in the upper diagram and surfactin production in the lower diagram, for the strains KM1016 (black square), CT10 (black circle), CT11 (gray circle) and CT12 (white circle).

References

Hoffmann M, Braig A, Fernandez Cano Luna DS, Rief K, Becker P, Treinen C, Klausmann P, Morabbi Heravi K, Henkel M, Lilge L, Hausmann R (2021) Evaluation of an oxygen-dependent self-inducible surfactin synthesis in *B. subtilis* by substitution of native promoter P_{srfA} by anaerobically active P_{narG} and P_{nasD}. AMB Expr 11:57. https://doi.org/10.1186/s13568-021-01218-4

Morabbi Heravi K, Altenbuchner J (2018) Cross talk among transporters of the phosphoenolpyruvate-dependent phosphotransferase system in *Bacillus subtilis*. J Bacteriol 200:e00213-18. https://doi.org/10.1128/JB.00213-18

Motejadded H, Altenbuchner J (2007) Integration of a lipase gene into *Bacillus subtilis* chromosome: Recombinant strains without antibiotic resistance marker. Iranian Journal of Biotechnology 5:105-109

Rahmer R, Morabbi Heravi K, Altenbuchner J (2015) Construction of a super-competent *Bacillus subtilis* 168 using the P_{mtlA} *-comKS* inducible cassette. Front Microbiol 6:1431. https://doi.org/10.3389/fmicb.2015.01431
